# Evaluation of a Novel Antibiotic Teaching Resource

**DOI:** 10.1007/s40670-020-00927-y

**Published:** 2020-02-11

**Authors:** Angharad P. Davies

**Affiliations:** Institute of Life Science, Swansea University Medical School & Public Health Wales, Singleton Park, Swansea, SA2 8PP UK

**Keywords:** Antimicrobials, Antimicrobial resistance, Teaching, Educational methods, Evaluation

## Abstract

Antimicrobial resistance presents a major challenge for healthcare and education of future prescribers is critical. Integrated medical courses allow more limited time for teaching the science of clinical microbiology, which underpins antimicrobial prescribing, making this a difficult topic for students. An innovative educational resource based on a game was created and evaluated in medical student teaching. Most students reported that the game assisted learning. However, testing showed that recall did not improve after using the resource. Student perceptions of resource efficacy may not correlate with test scores. The longer-term positive effect of enhanced student engagement is more difficult to measure.

## Background

Antimicrobial resistance (AMR) is one of the major challenges facing modern healthcare. Indeed the World Health Organization and World Bank cite this as one of the biggest crises facing the world today [1, 2]. When considering prescribing any drug, a balance must be struck between the likely benefit to the patient and the possible harm in terms of toxicity. With antibiotics, there is an additional consideration, namely the interests of society and future patients, in terms of resistant organisms that might result. This makes antibiotic prescribing uniquely complex and education of prescribers is vital in order to ensure proper antimicrobial stewardship (AMS).

For a proper understanding of antimicrobial use, some underpinning knowledge of basic clinical microbiology is required. Unfortunately, the profound pressure on teaching time in the medical curriculum, especially in 4-year graduate-entry courses, means that in many medical schools, it is difficult to find sufficient time for clinical science teaching. A survey of UK medical schools carried out in 2014 by the British Society for Antimicrobial Chemotherapy found that time devoted to antimicrobial-related teaching ranged between as few as 12 and up to 249 h out of 5000-h teaching time in a typical 5-year course [3]. As a result of this and the complexity of the subject, antibiotic prescribing is an area that students and junior doctors particularly struggle with. New and engaging ways of teaching are necessary to better teach this complex area of medicine in a crowded curriculum.

‘Top Trumps’® is a popular children’s card game now available commercially on myriad themes, from Dinosaurs to Harry Potter. Its precursor, ‘Quartets’ was originally devised in the 1960s as an educational tool for children. Success at the game relies on remembering the relative scores of various features of each item (e.g. *Tyrannosaurus Rex* scores highly for ferocity and *Diplodocus* scores low).

A set of antibiotic-themed ‘Trumps’ cards was created for use in healthcare education, and an evaluation carried out of their efficacy as a teaching tool for medical students.

## Activity

**Fig. 1:**
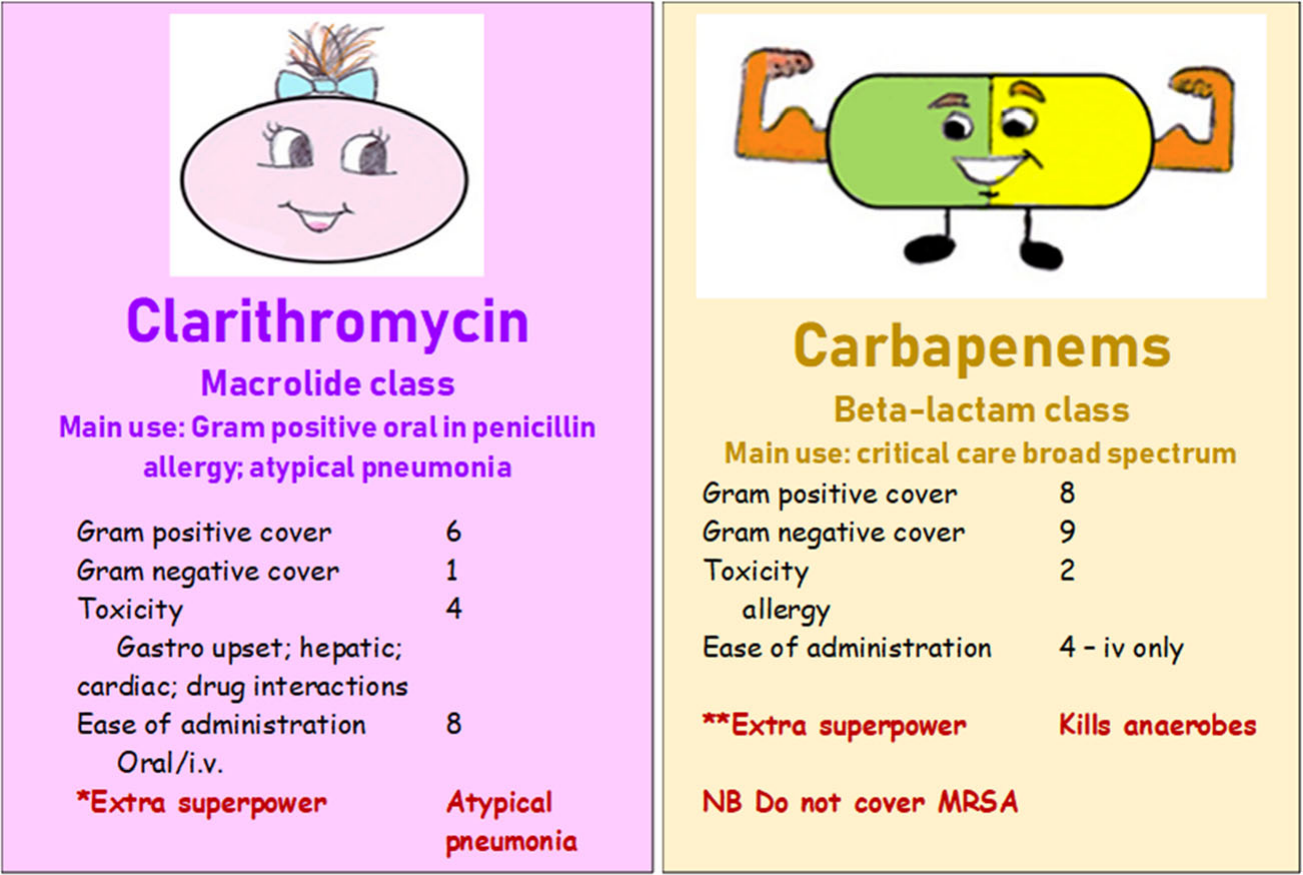
Example cards

A game in the style of ‘Top Trumps’®, based on antibiotics, was created. A range of commonly used antibiotic agents were included (penicillin, flucloxacillin, co-amoxiclav, clarithromycin, ciprofloxacin, vancomycin, gentamicin, cephalosporins, and carbapenems). Each card represented an antibiotic, with scores ascribed for Gram-positive activity, Gram-negative activity, toxicity, and ease of administration. Extra ‘superpowers’ (e.g. ‘covers anaerobes’) and useful facts (e.g. ciprofloxacin is an oral agent which can cover *Pseudomonas* sp.) were also added where relevant. Cards were colour coded so that all antibiotics from the same class (e.g. beta-lactams) had the same background colour. Two example cards are shown in Fig. 1.

An optional antibiotic tutorial was organised for first-year medical students on a graduate-entry medical course. Around 60 students attended (over half the year group of 96), reflecting students’ recognition of this as an important subject. At this stage, students had learned about antimicrobial resistance and stewardship generally but had not had teaching on individual agents, so no prior knowledge of the topic was assumed. The students were given a standard slide presentation with basic information about antibiotics, covering the same material as was included on the Trumps cards. The students were then asked to complete a short written test to assess their recall of the factual content (example questions: ‘Name an antibiotic that covers anaerobes’; ‘Name an antibiotic that can be used to treat atypical pneumonia’). They were then provided with a set of antibiotic Trumps per pair of students and asked to play with them for 10 min. The rules were explained for those students unfamiliar with them. The students then completed a second short written test, containing similar but different questions judged to be of equivalent difficulty. Since the Trumps were untested, the presentation and game were presented consecutively (rather than splitting the group into two cohorts with different interventions) so that all students benefitted from the same opportunity to learn. Thirty-six students completed all aspects of the activity and submitted a complete dataset (the rest submitted incomplete data).

Written student feedback comments were also collected. The evaluation was carried out in early 2019.

## Results and Discussion

**Fig. 2:**
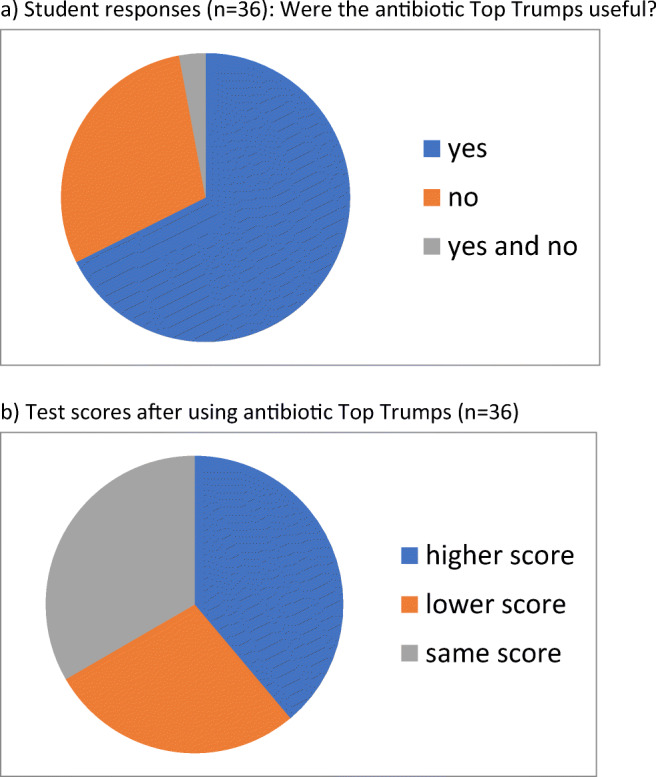
**a** Student responses (*n* = 36): Were the antibiotic Top Trumps useful? **b** Test scores after using antibiotic Top Trumps (*n* = 36)

Results are summarised in Fig. 2. When asked whether or not they found the game useful for learning, 23/36 (64%) said that they did. However in terms of the test scores, there was little difference before and after playing with the cards. A total of 14/36 (39%) scored slightly higher after playing, 10/36 (28%) scored slightly lower after playing, and 12/36 (33%) had unchanged test scores. Overall across all students, there was an increase in the combined test scores after playing with the cards from 136 to 145—a 6% improvement across all the students, equivalent to a 0.17% improvement for each student when averaged out.

Comments from students who believed the game was useful for learning often contained the theme that it was fun and also included ‘The more learning methods the better’, ‘Really liked them, thought the numerical Gram positive/Gram negative rating was helpful and the pictures were nice’, and ‘Fun very useful way to apply knowledge – would love to have them available for myself’. Three students commented that they would be good revision aids.

Comments from students who did not think the game was useful for learning included ‘Fun but not useful to learn’, ‘Too fast paced to take anything in properly’, ‘Focus too much on numbers when playing and not actual cards’, and ‘Wasn’t really paying attention to what drug was on each card, just the stats’.

Education has a fundamental role to play in combating antimicrobial resistance and promoting AMS, as outlined in the recently published UK national 5-year action plan [4]. Education in AMR concerns not only doctors but other independent prescribers, antimicrobial pharmacists, physician associates, nursing staff, and others who are involved in stewardship activities. Public Health England has published antibiotic prescribing and stewardship competencies for UK prescribers [5] and this was later followed by a set of competencies for undergraduate healthcare students [6]. A gap analysis by Health Education England found that the coverage of these principles in the education of various professional groups is suboptimal [7]. In terms of the medical student undergraduate curriculum, the introduction of the GMC (General Medical Council)’s Medical Licensing Assessment is a potential opportunity to ensure that education in AMR and AMS is further prioritised.

Students participating in this interactive activity were clearly engaged with it and found the exercise enjoyable. However, they tended to over-estimate how effective it had been at improving recall. With the time constraints of the medical curriculum, innovative approaches are to be welcomed but need to be evaluated to ensure they produce the required outcome in terms of learning. The activity described here is primarily suitable for use as an ice-breaker, or to break up more didactic teaching sessions, or as a revision aid.

Student perceptions of the efficacy of an intervention may not correlate with test scores. However, there is an additional value which is more difficult to measure in terms of enhancing student enjoyment and engagement with a difficult topic. In addition, it is highly plausible that playing with the cards at intervals over weeks would result in better retention of information. Funding has now been received to produce a large number of sets of cards professionally. This will enable them to be distributed to the students for use over a longer time period and future work will examine the impact of this.
